# Breaking the News or Fueling the Epidemic? Temporal Association between News Media Report Volume and Opioid-Related Mortality

**DOI:** 10.1371/journal.pone.0007758

**Published:** 2009-11-18

**Authors:** Nabarun Dasgupta, Kenneth D. Mandl, John S. Brownstein

**Affiliations:** 1 Department of Epidemiology, Gillings School of Global Public Health, University of North Carolina, Chapel Hill, North Carolina, Unites States of America; 2 Children's Hospital Informatics Program at Harvard–Massachusetts Institute of Technology, Division of Health Sciences and Technology, Boston, Massachusetts, United States of America; 3 Division of Emergency Medicine, Children's Hospital Boston, Boston, Massachusetts, United States of America; 4 Department of Pediatrics, Harvard Medical School, Boston, Massachusetts, United States of America; University of Queensland, Australia

## Abstract

**Background:**

Historical studies of news media have suggested an association between reporting and increased drug abuse. Period effects for substance use have been documented for different classes of legal and illicit substances, with the suspicion that media publicity may have played major roles in their emergence. Previous analyses have drawn primarily from qualitative evidence; the temporal relationship between media reporting volume and adverse health consequences has not been quantified nationally. We set out to explore whether we could find a quantitative relationship between media reports about prescription opioid abuse and overdose mortality associated with these drugs. We assessed whether increases in news media reports occurred before or after increases in overdose deaths.

**Methodology/Principal Findings:**

Our ecological study compared a monthly time series of unintentional poisoning deaths involving short-acting prescription opioid substances, from 1999 to 2005 using multiple cause-of-death data published by the National Center for Health Statistics, to monthly counts of English-language news articles mentioning generic and branded names of prescription opioids obtained from Google News Archives from 1999 to 2005. We estimated the association between media volume and mortality rates by time-lagged regression analyses. There were 24,272 articles and 30,916 deaths involving prescription opioids during the seven-year study period. Nationally, the number of articles mentioning prescription opioids increased dramatically starting in early 2001, following prominent coverage about the nonmedical use of OxyContin. We found a significant association between news reports and deaths, with media reporting preceding fatal opioid poisonings by two to six months and explaining 88% (p<0.0001, df 78) of the variation in mortality.

**Conclusions/Significance:**

While availability, structural, and individual predispositions are key factors influencing substance use, news reporting may enhance the popularity of psychoactive substances. Albeit ecological in nature, our finding suggests the need for further evaluation of the influence of news media on health. Reporting on prescription opioids conforms to historical patterns of news reporting on other psychoactive substances.

## Introduction

Historical studies of news media have suggested a strong association between media reporting on psychoactive substances and a subsequent increase in their use and abuse. Period effects for drug abuse have been documented for different classes of legal and illicit substances, with extensive research documenting the role that media publicity may have played in their emergence, regression and public perception [1], [2]: amphetamine [3] in the 1950s; glue sniffing [4] and methamphetamine [3] in the 1960s; fentanyl [5], crack and cocaine [6]–[11] in the 1980s; methamphetamine [3], methcathinone [5], Rohypnol® [5], and ecstasy [5], [12], [13] in the 1990s; and OxyContin® [14] and other prescription opioids [15], [16] in this decade.

The roles that news media may play in drawing awareness to national drug problems has been the subject of scrutiny in sociology for decades. Many researchers have examined the content of news media coverage of psychoactive substance use. In their commentary on the role of media in shaping ideas about health Caburnay et al. (2003) point out that, “news media are an important and influential part of the social environment, calling attention to certain issues by the amount and nature of their coverage.” [17] After reviewing decades of public opinion surveys regarding drug policy, Blendon and Young (1998) concluded that the American public's views “are largely shaped by the content and magnitude of media coverage on the issue.”[18] Despite these observations and the extensive qualitative analysis of the manner in which drug use is covered by the media, there has been little attempt to quantify the relationship between media reporting and drug use outcomes using epidemiologic techniques. Recent advances in technology allow us to harness the electronic news sources to uncover the temporal relationship between media reporting and substance abuse indicators to lend support to qualitative analysis conducted by others about reporting on drug use [4], [5], [8]–[10], [19], [20].

While availability and individual predispositions influence initiation of substance use, elements of the social environment, including news reporting may enhance the popularity of psychoactive substances among potential nonmedical users and foster demand. Even derogatory media reports may raise awareness and pique the curiosity of those inclined to experimentation. Some researchers have suggested that fear-based media messaging may contribute to decreases in drug use [21], but the temporal sequencing of drug problems and media reporting has not been well documented. If news articles only reported on already existing problems, and were a result and not a cause of adverse drug events, we would expect to first see an increase in substance abuse indicators, followed by an increase in the number of media reports mentioning the drugs. Alternatively, if media reports precede a substance abuse indicator, there may be a more complex relationship in which the media somehow influence drug abuse trends [2]. Here, we sought to quantify the temporal association between media reports and opioid-related mortality.

## Methods

### Mortality Data

We generated a monthly time series of unintentional poisoning deaths involving short-acting prescription opioid substances (including modified-release analgesics) in the US, from 1999 to 2005. We used multiple cause-of-death data published by the National Center for Health Statistics, accessed from the depository at the National Bureau of Economic Research. Unintentional prescription opioid poisoning deaths were identified using ICD-10 codes for poisonings of unintentional and undetermined intent (X42, X44, Y12), where short-acting pharmaceutical opioid substances were specified (T40.2, T40.4), and aggregated by month. Prescription opioid mortality data were used in this analysis because they are the only publicly available data source related to drug abuse with monthly data and national coverage. We limited the analysis to these years because more recent mortality data were not available at the start of the analysis, and previous years used a different coding classification for mortality which could have introduced an additional source of bias. We calculated population rates using mid-year Census estimates for each study year.

### News Media Data

Monthly counts of news articles mentioning generic and branded names of prescription opioids were obtained from Google News Archives from 1999–2005, accessed on October 16^th^, 2008. Media reports were retrieved using automated Perl scripts with manual verification [22]. Google News Archives provides access to English language print news media from about 25,000 sources. Month and year of publication and headlines were extracted for any article whose body text contained the generic or branded name of short-acting opioid substances available for outpatient use in the United States (from Food and Drug Administration's *Orange Book*): *buprenorphine*, *codeine*, *fentanyl*, *hydrocodone*, *hydromorphone*, *meperidine*, *propoxyphene*, *oxycodone*, and *tramadol*. *Morphine* was excluded because a preliminary review indicated articles mentioning *morphine* almost exclusively described pain management (without abuse) or arts reviews (e.g., the name of the band *Morphine*, storyline in the major motion picture *La Vie en Rose*). *Morphine* acted as a synecdoche for all opioid painrelievers used within a medical context. In order to establish a denominator to calculate rates of news articles, we established counts of all print news articles containing the word *the* in a given month.

We examined the subject material of articles which gave rise to each major peak in the time series of media reports by returning to the original news outlets. We present data on one branded (OxyContin) and one generic (fentanyl) opioid to demonstrate the types of events which drive news media volume. Articles were read and assessed by multiple authors for each time point. An automated text mining approach to classify articles was pilot tested, but is beyond the scope of this paper, and will be the subject of a future publication from our group. Results from manual article classification were used to identify the subjects of articles related to peaks in news volume.

### Statistical Analysis

We computed time-lagged correlations between the mortality time series and print news reports about short-acting opioid substances, compiled as described above. We used 5-month moving averages to smooth each time series, and performed lag regression to estimate the association between media volume and mortality rates. We assessed the relative predictive value of the time-lagged media data streams by fitting generalized linear models to mortality counts, assuming a Poisson distribution. We ran separate models where the predictor was the media time series, shifted up to 6 months after or 6 months before mortality. Overall model fit for each of the Poisson regression models was calculated by comparing deviance statistics with their asymptotic chi-square. The value of each lagged media series in predicting mortality was determined by calculating the proportion of the deviance explained, similar to the R^2^. Statistical models were run using Proc Genmod in SAS v9.1.2 (Cary, North Carolina).

Given the important geographic differences in opioid abuse in the United States [23] we also examined the state-level distribution of opioid-related news. Because of its dominant impact on news volume and recent concern [14], [24], we chose to only search the word *OxyContin* in conjunction with a mention of one of the 50 state names or the District of Columbia in the headline, body text or media source name. The natural language processing methods used in this analysis have been detailed elsewhere [25]. For the denominator, we found all articles for that year that contained the state name. We repeated this analysis for each year from 2000 to 2006 in order to calculate the state level proportions of news devoted to OxyContin. Graphical display of time-series maps are presented.

Mortality data used in this study were publicly available and de-identified, and the research was exempt from institutional review board oversight.

## Results

There were 30,916 deaths involving short-acting prescription opioids during the seven year study period, including deaths from toxic exposure to extended-release formulations containing short-acting opioid substances. Monthly short-acting pharmaceutical opioid-related mortality increased from 0.81 deaths per 1,000,000 US population in January of 1999 to 1.90 deaths per 1,000,000 US population in December of 2005. We also identified 24,272 articles mentioning prescription opioids among a total of 31,651,000 articles queried in Google News Archives from 1999–2005 (0.08% of all articles).

**Figure 1:**
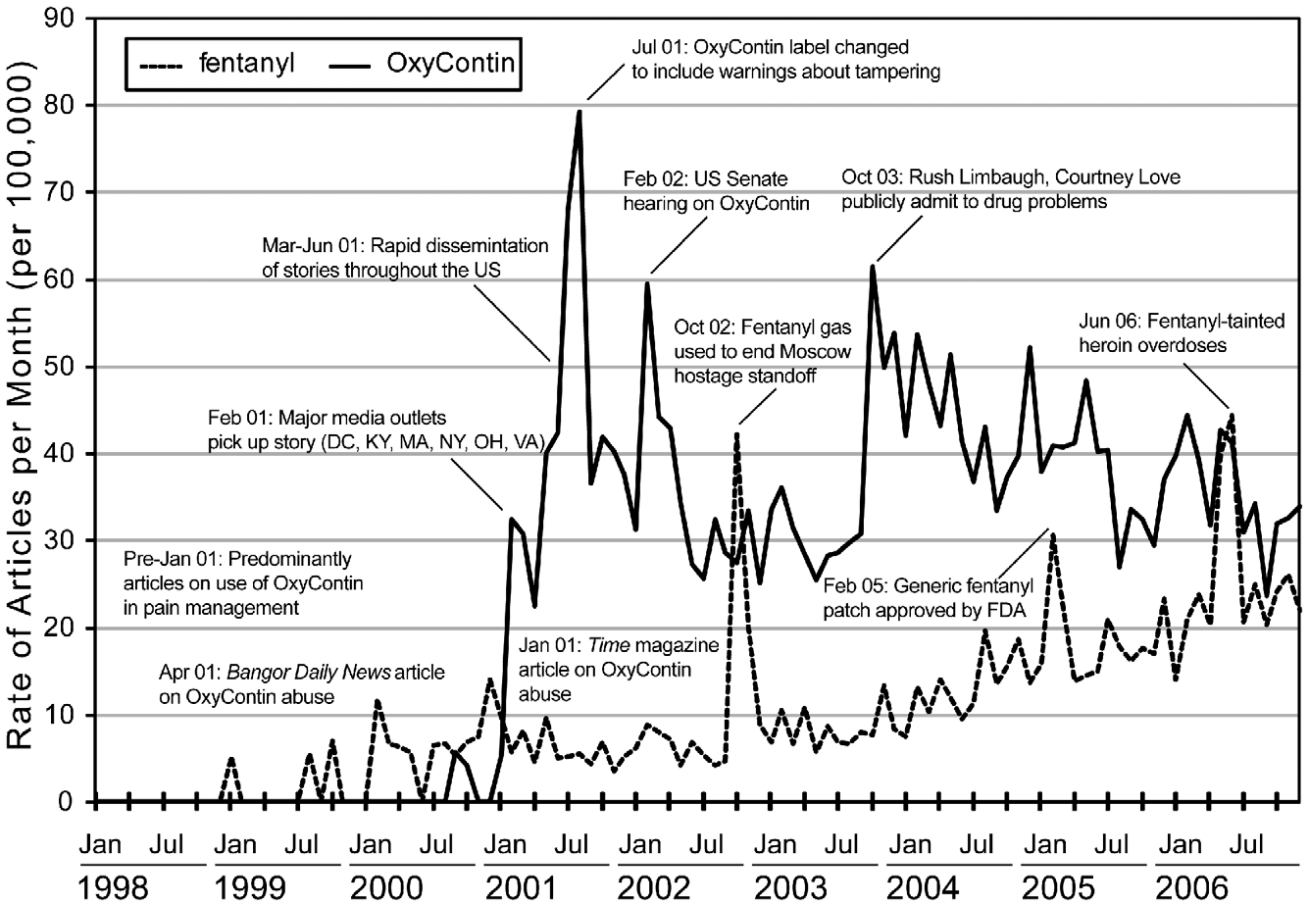
Monthly rates of news volume mentioning prescription opioids, 1998–2006, United States. Monthly print news volume mentioning prescription opioids was driven by different factors over time. Government and industry actions and celebrity involvement tended to produce peaks in the time series. A general pattern was observed of regional reports of abuse problems followed by national coverage, for OxyContin. By comparison, fentanyl was reported on less frequently than OxyContin. Two peaks in fentanyl articles were due to issues not related to pharmaceutical fentanyl formulations (weaponized gas and illicitly manufactured powder), suggesting the need for categorization of articles based on content. Abbreviations: Apr, April; DC, District of Columbia; Feb, February; FDA, Food and Drug Administration; Jan, January; Jul, July; KY, Kentucky; MA, Massachusetts; Mar, March; NY, New York; Oct, October; OH, Ohio; US, United States; VA, Virginia.

Over time the most common articles were about individuals arrested for drug selling possession (diversion) and those articles raising general concerns about prescription opioid abuse, dependence and poisoning, as has been noted by others [24]. Manual review of stories about prescription opioids revealed that time series peaks in article volume were related to celebrities using drugs, federal government hearings, and pharmaceutical company actions (Figure 1), similar to what was documented for coverage on cocaine use during the mid-1980s [26]. Medical licensing decisions censuring health care workers, pharmacy robberies and recovery from drug dependence less commonly contributed to peaks in news volume, but were reported at consistent rates over time (data not shown). Articles about prescription opioid overdoses tended to report on arrests and criminal proceedings against those accused of being involved in providing the drugs to the decedents, including doctors, drug dealers and family members. Overdose articles tended to focus on younger decedents, despite the average age for prescription opioid poisoning being in the late 30 s in the United States during this time period [23]. Peaks were also generated due to spurious issues involving opioids, such as the use of fentanyl by Russian authorities to end a hostage situation in a Moscow theatre in October 2002 and illicitly-manufactured fentanyl-tainted heroin in the Summer of 2006 (Figure 1).

**Figure 2:**
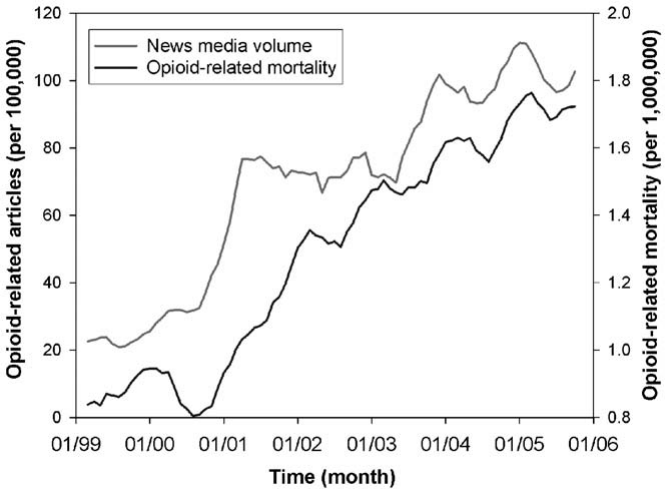
Opioid-related news media reports and poisoning mortality, 1999–2005, United States. Monthly print news media volume mentioning short-acting prescription opioid substances closely tracked closely with poisoning mortality due to those medications. Print news media articles consistently preceded mortality by a few months. Month and year of publication and headlines were extracted for any article whose body text contained the generic or branded name of short-acting opioid substances available for outpatient use in the United States during the study period: *buprenorphine*, *codeine*, *fentanyl*, *hydrocodone*, *hydromorphone*, *meperidine*, *propoxyphene*, *oxycodone*, and *tramadol*. *Morphine* was excluded because a preliminary review indicated articles mentioning *morphine* almost exclusively described pain management (without abuse) or arts reviews. Poisoning deaths were identified using a combination of International Classification of Disease 10^th^ Edition (ICD-10) codes, see text, to identify deaths involving short-acting opioid substances. Data on methadone are not presented due to difficulties in distinguishing deaths from its two indications (addiction, pain) using ICD-10 codes, however inclusion of methadone did not significantly alter results (data not shown). Time series were smoothed using 5-month moving averages.

**Figure 3:**
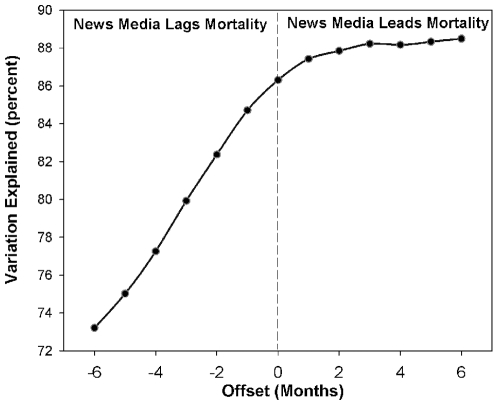
Time delay between news reports and poisoning mortality, by month, 1999–2005, United States. Print news media reports mentioning short-acting opioid substances consistently preceded poisoning mortality from those medications, with peak correlation at 5 to 6 months before mortality. In this cross-correlation analysis we assessed the relative predictive value of the time-lagged media data streams by fitting generalized linear models to mortality counts, assuming a Poisson distribution. We ran separate models where the predictor was the media time series, shifted up to 6 months after or 6 months before mortality. Overall model fit for each of the Poisson regression models was calculated by comparing deviance statistics with their asymptotic chi-square. The value of each lagged media series in predicting mortality was determined by calculating the proportion of the deviance explained, similar to the R^2^. The greatest percent of variation explained was highest in scenarios where the news media preceded mortality.

The number of articles mentioning prescription opioids increased by 149% from January 2001 to July 2001, following prominent national coverage about the nonmedical use of OxyContin® (Figure 2). We found news media to be a significant predictor of mortality (*p*<0.0001, df 78), even after adjusting for effects of seasonality and linear trending. We performed lagged-regression analysis to quantify a temporal relationship between mortality and new reporting (Figure 3). News reports preceded poisoning deaths with peak correlation at 2–6 months prior to mortality, accounting for 88% of the variability.

**Figure 4:**
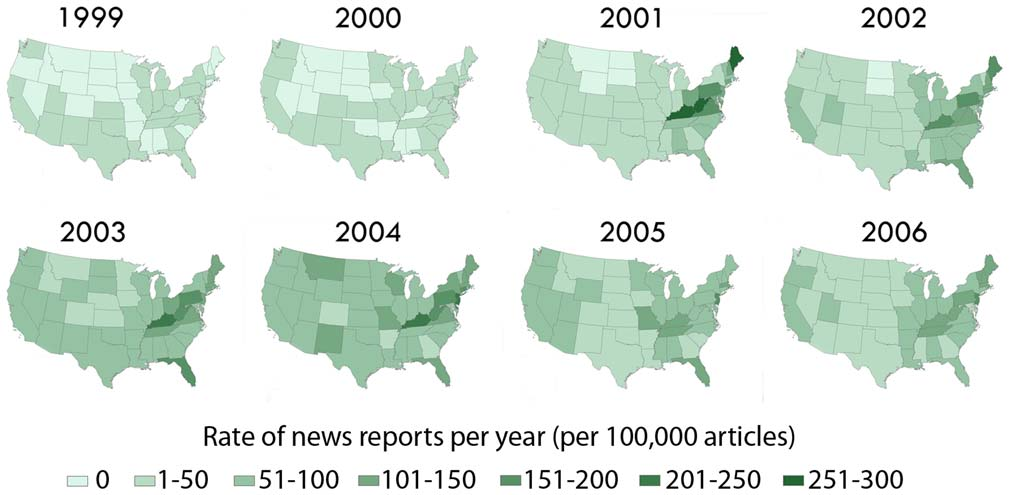
Rates of OxyContin print news media mentions in the United States, 1999–2006, by state. Print news media articles showed a progression from localized reporting, to national attention and eventual regression between 1999 and 2006, based on an analysis where articles were geotagged based on the US state which the article was describing or was published in, see text for methodology. OxyContin problems were first described in 2001 with particular focus on the Appalachian region and New England. Between 2001 and 2003, there was a sharp increase in articles about OxyContin published about Florida, Pennsylvania and New Jersey. The highest state-level rates of OxyContin news articles were in 2001. After 2003, reporting became more homogenous with substantial reporting throughout the country. Media attention on OxyContin started to subside soon afterwards, with a return to localized reporting in 2005. By 2006, New Jersey had the highest rates, as articles about abuse- and tamper-resistant formulations in development become prominent; New Jersey has a large concentration of pharmaceutical manufacturers and many of these articles were from financial news sources describing attempts to develop formulations that would be less prone to tampering. The geo-temporal spread of news reports about OxyContin mirror the experiences of previous cycles of drug abuse panics in the United States.

We found important differences in the geographic distribution of OxyContin-related news articles (Figure 4). Prior to 2001 when the focus on articles about OxyContin was on pharmaceutical earnings and pain management, reporting was highest in New Jersey, a state with a high concentration of pharmaceutical companies and businesses providing financial and supporting services to the industry. In 2001–2003, increases in the proportion of news articles about OxyContin expanded throughout the Appalachian region and New England, with foci eventually developing in Florida, Pennsylvania and New Jersey. After 2003, reporting became more homogenous with substantial reporting throughout the country. By 2006, New Jersey had the highest rates as articles about abuse- and tamper-resistant opioid pain reliever formulations became prominent. Similar cycles have of the media reporting on industry attempts to create less abuseable forms have been noted by others [4].

## Discussion

Our finding that articles about prescription opioids routinely preceded overdose deaths associated with the same medications supports previous research, but we cannot draw a causal conclusion from our study alone, as other researchers have pointed out [12]. In conjunction with the work of others [2], [3], [8], [9], [26], our findings support previous hypotheses about role of the news media in perpetuating public beliefs about psychoactive substance use, and the potential role the media may have in drug use trends. We document that reporting on prescription opioids in the 2000s followed strikingly similar patterns as for drugs in previous decades, and we provide a quantitative argument supporting the often-repeated demand for journalistic standards for reporting on substance use issues.

The pattern of news media outlets reinforcing each other to arrive at a general held belief has been noted for stories about epidemics of other drugs of abuse [3], [5], [8], [9], [27 [cited in 11]]]. This phenomenon is termed *intermedia convergence* by Reese and Danielian (1989), and has been recently noted to be accelerated in the current more rapid and internet-influenced news cycle [28]. The early emergence of problems as reported by the news media about northern New England and Appalachia may in part explain the sustained focus on these regions (see [29]), despite national level data suggesting a broader ranging problem [30]. In a recent review of media portrayals of drug use, Talyor (2008) cites extensive sociological evidence to advance the theory that news reports have focused on “outsiders,” socially marginalized classes of people, often racial minorities, going as far back as the mid-1800s in the United States [see also: 19]. In this paper we document that with prescription opioids in the current decade, the outsiders appear to be rural residents, with a particular focus on Appalachia and northern New England, as shown in Figure 4.

We posit that there may be co-linear relationships between the medical use of a prescription opioid, medical consequence from the use and abuse of the product, the public attention devoted to the topic, and news media reports. Co-linearity does not prove causation, however, and our intention in conducting this research is to advance the dialogue about the potential interconnectedness of these seemingly independent phenomena. While news articles may contribute indirectly to illicit demand for short-acting prescription opioids, there are many complex factors that influence poisoning mortality and are poorly understood: improper marketing, lax prescribing standards, patient and health care worker errors, breaches of the pharmaceutical supply chain, the influence of social networks, individual physical comorbidities, intervention programs and fear of accessing emergency medical care.

Clinical experience leads us to believe that drug use and overdose have multiple influences at the individual, social and genetic levels. The same multi-factorial influences may make drug abuse stories attractive subjects for journalists, combined with the fundamental issues crossing sociodemographic lines. It has also been noted that “media portrayals of the consumption and effects of alcohol do not comprise a neutral, scientific reality but rather signify a network of social knowledge, value systems, and symbols,” with the same likely occurring for other substances [31]. There may be public expectation for coverage, and while predictable, the media aren't necessarily acting beyond the pale of their professional responsibilities. Yet, there is little formal guidance to journalists for appropriate coverage of this sensitive topic. The editors of PLoS point out that “today's health reporters may have been covering crime last week and politics the week before,” [32] and media reporting of medical news has long been criticized [33]. At the intersection of crime, health and politics, news reporting about drug abuse reflects a complex interaction of social factors, with little guidance as to how these issues should be covered. A recent article by PLoS Medicine editors on news coverage of suicides in the military reminds us that “reporters have a choice about how they frame stories about mental illness” [34]. While individual-level influence of news reporting cannot be deduced from surveillance-oriented studies, and require more intensive analyses [35], [36], our results suggest the need for a thorough evaluation of this topic, with an eye towards ethical standards that promote responsible journalism [12]. These standards could be analogous to guidelines for stories on suicide [37]–[40], where the role of the news media in copycat suicides has been well documented [39], [41]–[43], although some researchers believe the link to be less than definitive [44]. Furthermore, our study suggests that there may be opportunity to harness news reports as an early warning indicator of emerging drug problems, similar recent work in infectious disease surveillance [25].

In support of the long-standing need for journalistic standards, we identified certain articles that amounted to inadvertent endorsements of prescription drug abuse; while dangers associated with drug abuse were mentioned in later paragraphs of articles, the opening lines presented an enticing scenario. A 2000 *Portland Press Herald* article opened with: “Euphoria envelopes your body in a warm, cozy hug. Problems dissolve. Limbs tingle. Life feels perfect. These are the sensations that drug addict Christopher Coughlin says he felt using OxyContin, a highly addictive opiate that is sweeping Maine, from the streets of South Portland to the rural communities of Washington County.” [45] Strikingly, Breecher (1972) noted nearly identical language in a *Newsweek* article from 1962, suggesting a longstanding cyclical pattern in the way news about drug abuse is presented: “You're in outer space. You're Superman. You're floating in air, seeing double, riding next to God. It's Kicksville. Are these the fantasies of narcotics addicts on a pop? No. More disturbingly, these hopped-up reactions are those of teenagers hooked on goofballs, model airplane glue, and cough medicine. Across the nation, police last week reported case after case of this alarming trend.” [4], [46] The similarity of language goes beyond semantics; it suggests a consistent mindset and approach to reporting about substance use that has not changed substantially in at least four decades, if not longer [47].

Our findings are consistent with findings from previous cycles of drug abuse concern. At the height of reporting on cocaine during the summer of 1986, Merriam (1989) found that 5% of space in national media were devoted to drug issues, starting at less than 1% in the start of that year [8]. By using electronic news sources, we were able to expand the analytic space to include local and regional print news media. Reflecting the greater volume and diversification of news media outlets in the 2000s, we found that only 0.08% of news volume involved prescription opioids. While we cannot make a direct comparison between previous work and our analysis, new media will have to be considered in future analyses. Print news media articles serve as a measureable proxy for other forms of media that may also mention prescription opioids. In today's rich multimedia environment competing and synergistic influences of multiple sources of information need to be assessed systematically, and future research may well focus on blogs and other electronic news sources. An analysis of internet comments about news reports of adolescent suicides suggested the strong influence of the style of reporting and attitudes about mental health [48]. Hawton and colleagues (1999) noted a short-term increase in patients presenting for intentional drug overdoses after the broadcast of a popular television drama depicting drug self-poisoning [49]. Basic health psychology research is needed to guide further efforts in this area.

We compared print news media articles to unintentional and undetermined intent poisoning mortality because vital statistics are the only publicly available data source with information on substance use disorders that has national coverage and can be analyzed on a monthly basis. Data from vital statistics reports are known to have limitations [50]–[52], with particular fears of underreporting. Medical examiners and coroners in the United States do not have standardized assessment and attribution procedures for suspected drug poisoning deaths. In addition, acute poisonings due to patients' medication dosing errors with potent opioids would be classified the same as a fatal opioid poisonings from drug abuse under ICD-10 coding. It is unknown what proportion of the deaths observed were from each type of exposure (and what the overlap may be), but deaths from medical errors involving specific prescription opioid pain relievers have resulted in nationwide alerts from the Food and Drug Administration [53], [54]. In order to address this problem we conducted sensitivity analyses using different combinations of ICD-10 codes representing wider and narrower definitions, such as including mental health codes as has been suggested by others [52]. While the absolute number of poisoning fatalities fluctuated with the different definitions, the proportional change appeared to be nearly constant over time, resulting in similar results in lag regression analysis. We do not present these results here for the sake of brevity.

Another possible explanation for part of the observed correlation is diagnostic suspicion bias, that medical examiners more carefully screened for opioid poisonings in deaths of uncertain cause, after they themselves were exposed the media reports about overdose, or concerns expressed in the medical literature. This type of response has not been noted for medical examiners and drug overdoses in the literature, but remains plausible. As our research team has described elsewhere [23], the proportion of unintentional poisoning deaths in which a narcotic was not specified decreased slightly in metropolitan areas between 1999 and 2003, and increased slightly in non-metropolitan areas. However it has been noted that voluntary reporting to the Food and Drug Administration's spontaneous adverse event reporting system increased following publicized problems with adverse events for pharmaceutical medications and devices [55]–[57]. To what extent this introduces bias in our analysis is unclear.

While previous generations of researchers have examined the question of the news media and substance use reporting, much of the findings have been sequestered to the sociology literature and have focused on qualitative analysis of media content. Few studies have attempted to link media data with information on drug use trends in a quantitative manner with temporal and geographic specificity. Substance use disorders have become increasingly medicalized in the last two decades (i.e., the “chronic relapsing brain disease” model), and pain has been more systematically assessed and aggressively treated with outpatient pharmacotherapies. Simultaneously there has been a profusion of the types of media involved in reporting news. However, biomedical science research has paid limited attention to the social context in which drug use problems and pain are perceived on a community level and what role mass media play, especially electronic sources. Returning to Blendon and Young (1998), “In the future, if health professionals want to change the direction of Americans' beliefs on particular drug policies they will have to devote significant resources to gaining media attention for their views.” We agree with them, but feel that journalism standards would also be beneficial. Perhaps the next “drug epidemic” will benefit from a more balanced and public health approach to reporting, but it requires immediate interdisciplinary action.
